# Origin and Evolution of Enzymes with MIO Prosthetic Group: Microbial Coevolution After the Mass Extinction Event

**DOI:** 10.3389/fgene.2022.851738

**Published:** 2022-03-29

**Authors:** Fei Peng, Ulrike Engel, Habibu Aliyu, Jens Rudat

**Affiliations:** Institute of Process Engineering in Life Sciences, II, Technical Biology, Karlsruhe Institute of Technology, Karlsruhe, Germany

**Keywords:** MIO-dependent enzymes, a mass extinction event, microbial coevolution, rooted phylogenetic tree, minimal ancestor deviation

## Abstract

After major mass extinction events, ancient plants and terrestrial vertebrates were faced with various challenges, especially ultraviolet (UV) light. These stresses probably resulted in changes in the biosynthetic pathways, which employed the MIO (3,5-dihydro-5-methylidene-4H-imidazole-4-one)-dependent enzymes (ammonia-lyase and aminomutase), leading to enhanced accumulation of metabolites for defense against UV radiation, pathogens, and microorganisms. Up to now, the origin and evolution of genes from this superfamily have not been extensively studied. In this report, we perform an analysis of the phylogenetic relations between the members of the aromatic amino acid MIO-dependent enzymes (AAM), which demonstrate that they most probably have a common evolutionary origin from ancient bacteria. In early soil environments, numerous bacterial species with tyrosine ammonia-lyase genes (TAL; EC 4.3.1.23) developed tyrosine aminomutase (TAM; EC 5.4.3.6) activity as a side reaction for competing with their neighbors in the community. These genes also evolved into other TAL-like enzymes, such as histidine ammonia-lyase (HAL, EC 4.3.1.3) and phenylalanine ammonia-lyase (PAL; EC 4.3.1.24), in different bacterial species for metabolite production and accumulation for adaptation to adverse terrestrial environmental conditions. On the other hand, the existence of phenylalanine aminomutase (PAM; EC 5.4.3.10) and phenylalanine/tyrosine ammonia-lyase (PTAL; EC 4.3.1.25) strongly indicates the horizontal gene transfer (HGT) between bacteria, fungi, and plants in symbiotic association after acquiring the PAL gene from their ancestor.

## Introduction

### MIO Prosthetic Group

**FIGURE 1:**
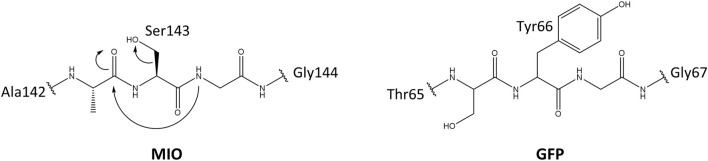
The proposed mechanism for the MIO in HAL and GFP formation by posttranslational modification.

Usually, the side chains of proteogenic amino acids act as nucleophiles in enzymatic catalysis. Due to the lack of strongly electrophilic groups, enzymes use metal ions and organic molecules to assist in electrophilic catalysis (Bischoff and Schlüter, 2012; Punekar, 2018). Besides cofactors from the environment, posttranslational modifications/conversions (PTMs) of amino acid side chains that provide a strongly electrophilic center are of the same importance in enzymes. (Müller, 2018). MIO (3,5-dihydro-5-methylidene-4H-imidazole-4-one) is such a catalytic moiety for the elimination of ammonia from arylalanine amino acids, which belong to arylalanine ammonia-lyase and aminomutase. This highly electrophilic moiety is spontaneously folded by an inner amino acid triad (Ala/Ser/Cys/Thr)-Ser-Gly. The glycine amide lone pair attacks the π* orbital of carbonyl in the amino acid, which is located at two positions preceding glycine. This nucleophilic attack is electronically unfavorable; therefore, mechanical compression from neighboring residues and connections with internal water molecules play a vital role by promoting backbone cyclization (Baedeker and Schulz, 2002; Sánchez-Murcia et al., 2016). The formation mechanism of MIO is similar to the chromophore in green (GFP) and red fluorescent protein (RFP) (Figure 1) (Reid and Flynn, 1997; Baedeker and Schulz, 2002).

**FIGURE 2:**
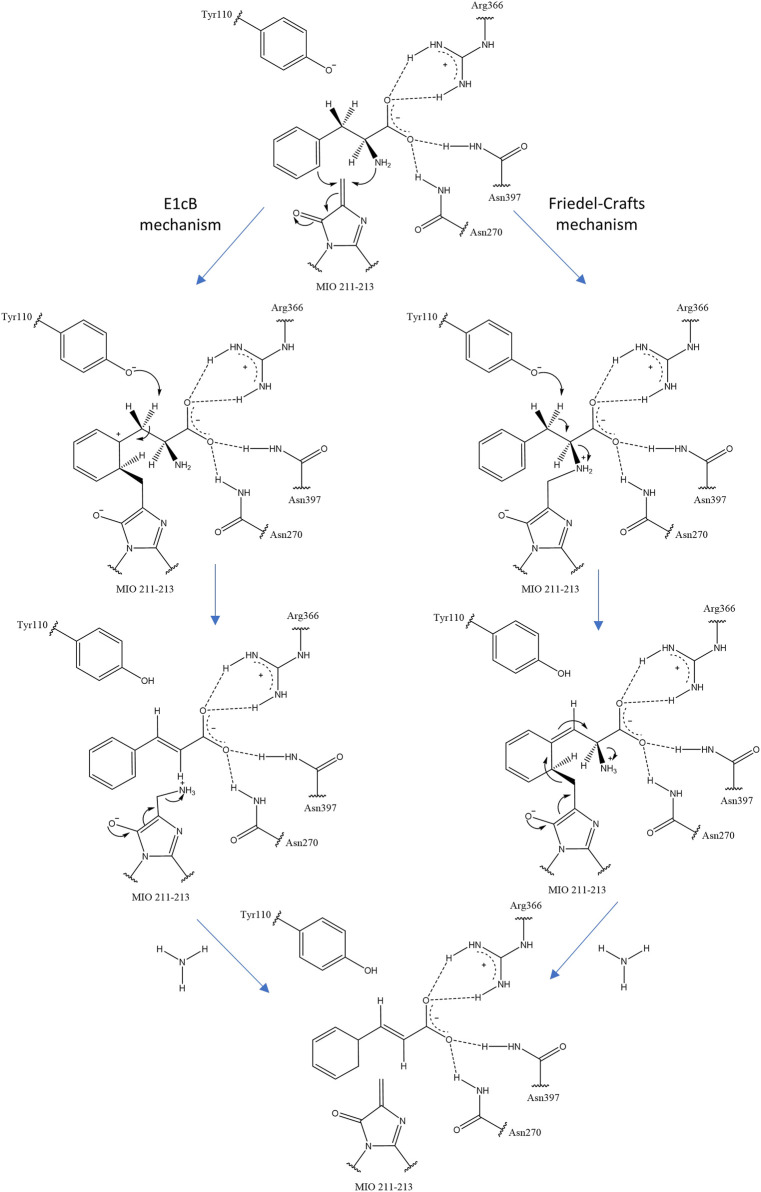
Two proposals for ammonia elimination by *R. toruloides* PAL. The other residues help to orient and stabilize the carboxyl group of arylalanine amino acids.

The MIO prosthetic group performs a nucleophilic attack on the substrate arylalanine amino acid, forming a covalent amino-MIO intermediate to enhance its acidity. In the most accepted E1cB mechanism of ammonia elimination in AAM, the intermediate binds the MIO at the benzylic position of the amino acid and is deprotonated by the enzymatic base. Then, the intermediate yields a carbanion intermediate and releases ammonia in the subsequent step. In the alternative mechanism (Friedel–Crafts), this reaction occurs at the aryl side chain of the substrate (Figure 2) (Punekar, 2018).

### Structures of MIO-dependent Enzymes

According to the crystal structure, the members of AAM are homotetramers, comprising four identical active sites that build among the residues of three interlocking monomeric subunits in a nose-to-tail way separately. Each monomer is composed of a rigid central core domain, a globular N-terminal domain, and an elongated C-terminal domain. In the N-terminal region, the MIO prosthetic group is located in a narrow tunnel, which is created by an inner and an outer loop. These two loops cap the tunnel and enclose the active site from solvents in HAL. However, they are more mobile in eukaryotic PAL, which restricts the access of the substrate and influences the mutase and lyase activity in MIO-dependent enzymes. The catalytic tyrosine residue (Tyr110 in *Rhodosporidium toruloides* PAL) on the inner loop is highly conserved in HAL, TAL, and PAL, which is necessary for catalytic activity since the mutants of this residue to alanine and phenylalanine are completely inactive (Röther et al., 2002; Wybenga et al., 2014). The residues in the carboxyl binding pocket promote the MIO prosthetic group formation and also interact with the substrate, representing essential binding sites in the active center (Wybenga et al., 2014), whereas the hydrophobic binding pocket provides sufficient space for the aromatic ring of the substrates and modulates the substrate specificity (Nagy et al., 2019).

### Phenylpropanoid Pathway

Replying to abiotic environmental stress, plants activate the phenylpropanoid pathway to accumulate phenolic secondary metabolites. As a major component of phenylpropanoids, flavonoids provide significant protective effects to plants in response to various unfavorable conditions (drought, heavy metals, salinity, and UV radiations) (Sharma et al., 2019). Photoprotection is the most important functional role of flavonoids. The epidermal flavonoids reduce protein and DNA damage by preventing dimerization of thymine, adsorbing radiations, and scavenging the reactive oxygen species (Kootstra, 1994; Treutter, 2006). Other specific metabolites, monolignols, confer tolerance to plant cell walls against chilling stress. The phenylpropanoid accumulation is regulated by the gene expression of corresponding enzymes in the biosynthesis pathway (Sharma et al., 2019).

**FIGURE 3:**
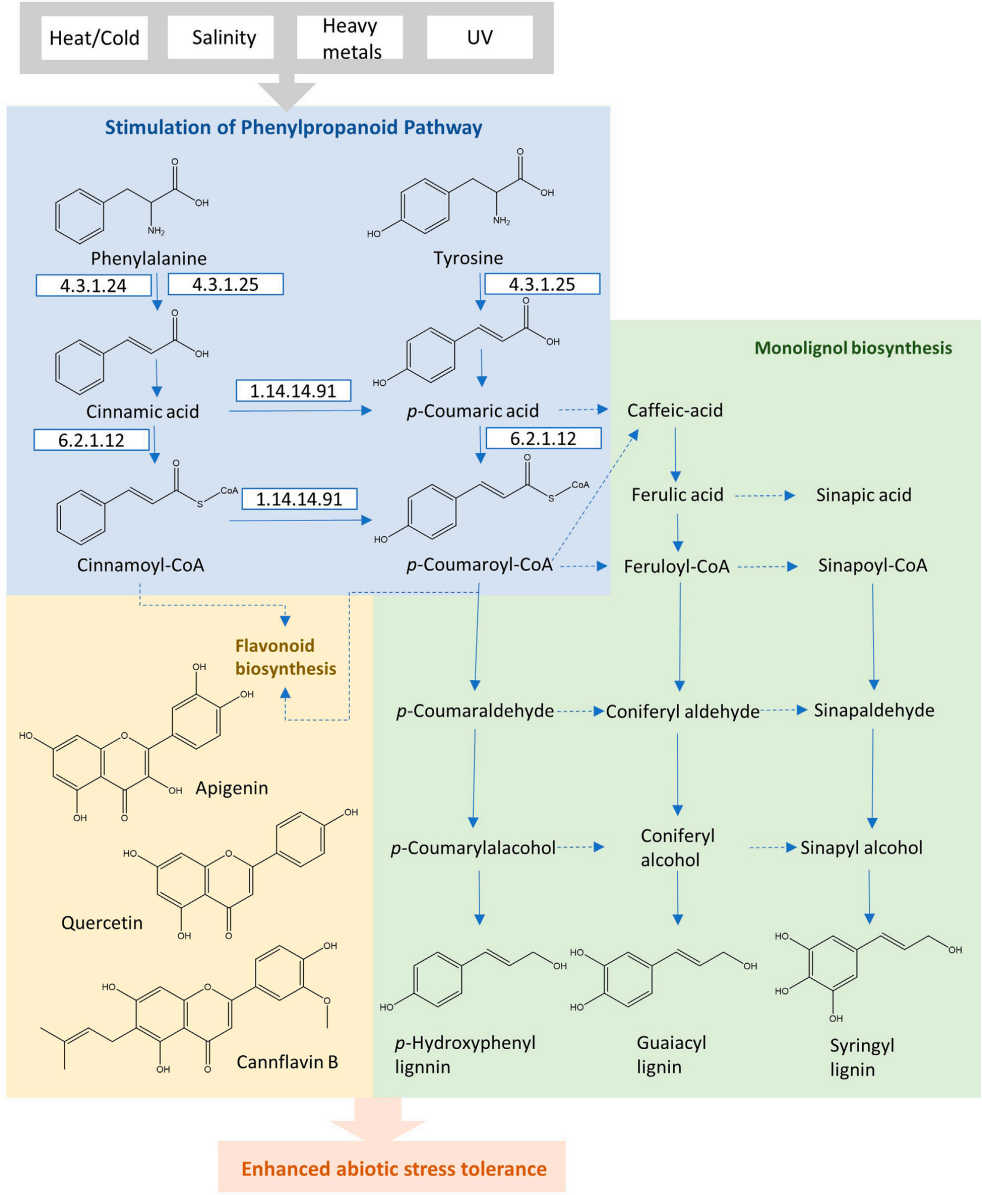
The phenylpropanoid pathway in plants for the production of flavonoids and monolignols under abiotic stress conditions.

PAL is the first regulatory enzyme that transforms l-phenylalanine into *trans*-cinnamic acid, controlling the carbon flux from the shikimate pathway to phenylpropanoid metabolism. The subsequent enzyme is cinnamic acid 4-hydroxylase (C4H; EC 1.14.13.11), which reduces *trans*-cinnamic acid, leading to the formation of *p*-coumaric acid. 4-coumaric acid-CoA ligase (4CL; EC 6.2.1.12) catalyzes the ATP-dependent formation of the *p*-coumaroyl CoA, which serves as the branch point in phenylpropanoid biosynthesis. The conversions by these initial three enzymes are necessary and form the main pathway skeleton in the higher plants. In general, l-phenylalanine is starting immediately from the shikimate pathway, and in certain monocot species, *p*-coumaric acid may directly be produced from l-tyrosine through PTAL with bifunctional activity, bypassing the hydroxylation by C4H (Figure 3). In comparison to dicotyledonous plants, these monocots form the cell walls with higher proportions of syringyl (S)-rich lignins, more esterified coumaric acid as well as flavonoid tricin (Barros et al., 2016). This preferential composition suggests the existence of alternative routes with PTAL in the lignin biosynthetic pathway. The methylation of caffeic acid in this parallel pathway employs the caffeate/5-hydroxyferulate 3-O-methyltransferase (COMT), encouraging an efficient conversion step of *p*-coumaric acid to caffeic acid through the cytosolic coumarate 3-hydroxylase (C3H) (Figure 3). This alternative pathway with PTAL and C3H in monocots bypasses the synthesis of H and G lignins by membrane-bound cytochrome P450, leading to the enhancing of (S)-lignin content efficiently (Barros et al., 2019).

### Histidine Pathway

**FIGURE 4:**
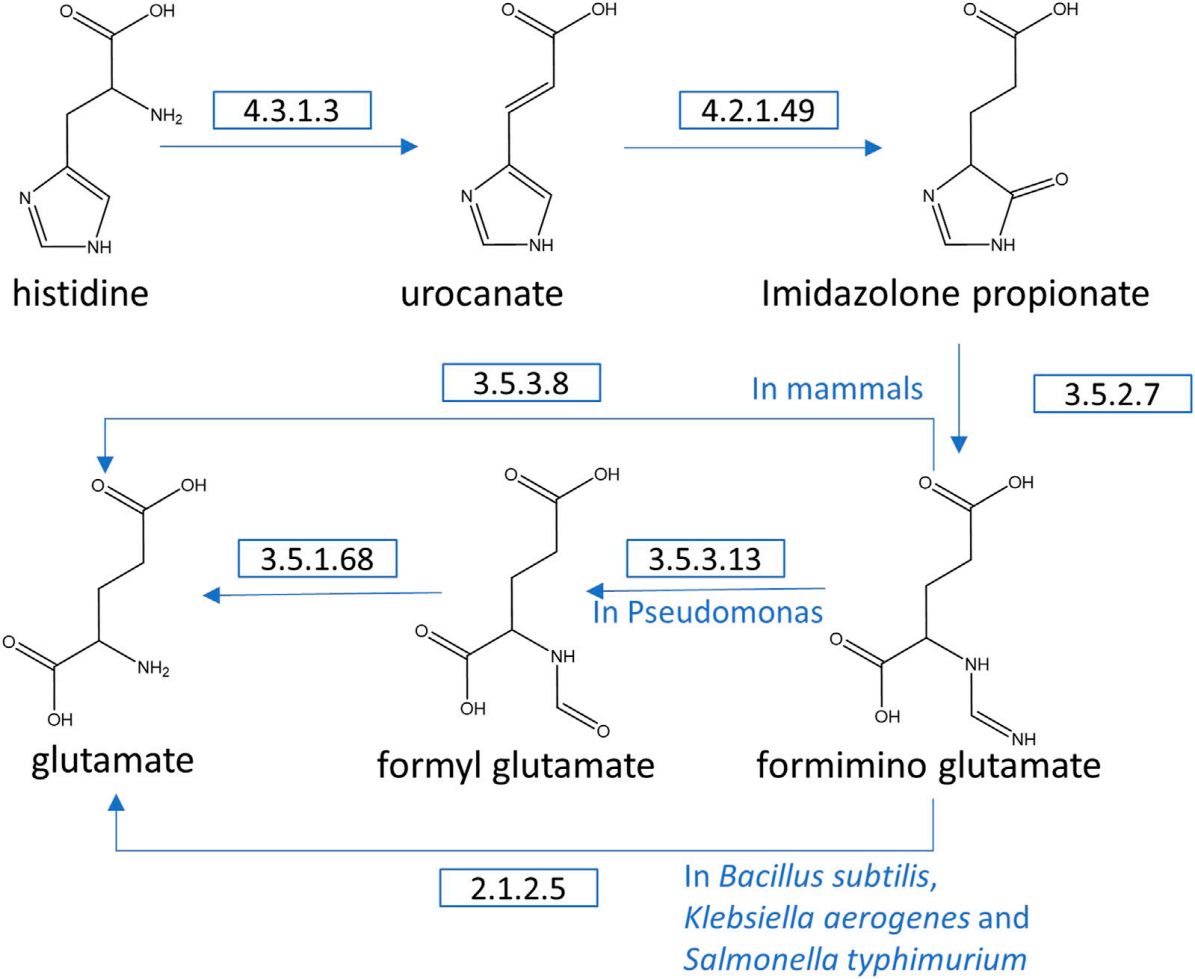
Different histidine degradation pathways. Conversion of histidine to glutamate.

Histidine catabolism is initiated by HAL, which is a universal enzyme to form trans-urocanic acid and release ammonia in the bacterial pathway. *trans*-Urocanic acid is transformed into 4-imidazole-5-propionate by urocanate hydratase (EC 4.2.1.49). Subsequently, the ring is cleaved by imidazolonepropionase (EC 3.5.2.7). Depending on the organism species, l-glutamate is generated by various enzymes from *N*-formimino-l-glutamate. In mammals, the *N*-formimino-l-glutamate is hydrolyzed by tetrahydrofolate (THF)-dependent glutamate formiminotransferase (EC 2.1.2.5). In bacteria, some genera (*Bacillus*, *Klebsiella*, and *Salmonella*) eliminate the formimino group in one step by formininoglutamase (EC 3.5.3.8), whereas the other genera, such as *Pseudomonas*, coregulate the *hutF* and *hutG* genes and express formimidoylglutamate deiminase (EC 3.5.3.13) and *N*-formylglutamate deformylase (EC 3.5.1.68) to yield the l-glutamate (Figure 4) (Bender, 2012; Kohlmeier, 2015).

HAL is crucial for growth in children, primarily in the skin and the liver. For example, one disease, histidinemia, results from a deficiency of HAL (Brosnan and Brosnan, 2020). The trans-urocanic acids, as UV-absorbing compounds, accumulate in the stratum corneum of the skin because of the absence of urocanase to eliminate them. Under UV light, *trans*-urocanic acid is isomerized into *cis*-urocanic acid until the quantity equation of these two isomers. The latter form probably initiates the immunoregulation under UV exposure (Norval, 2001).

## Results

### Phylogenetic Analysis

**FIGURE 5:**
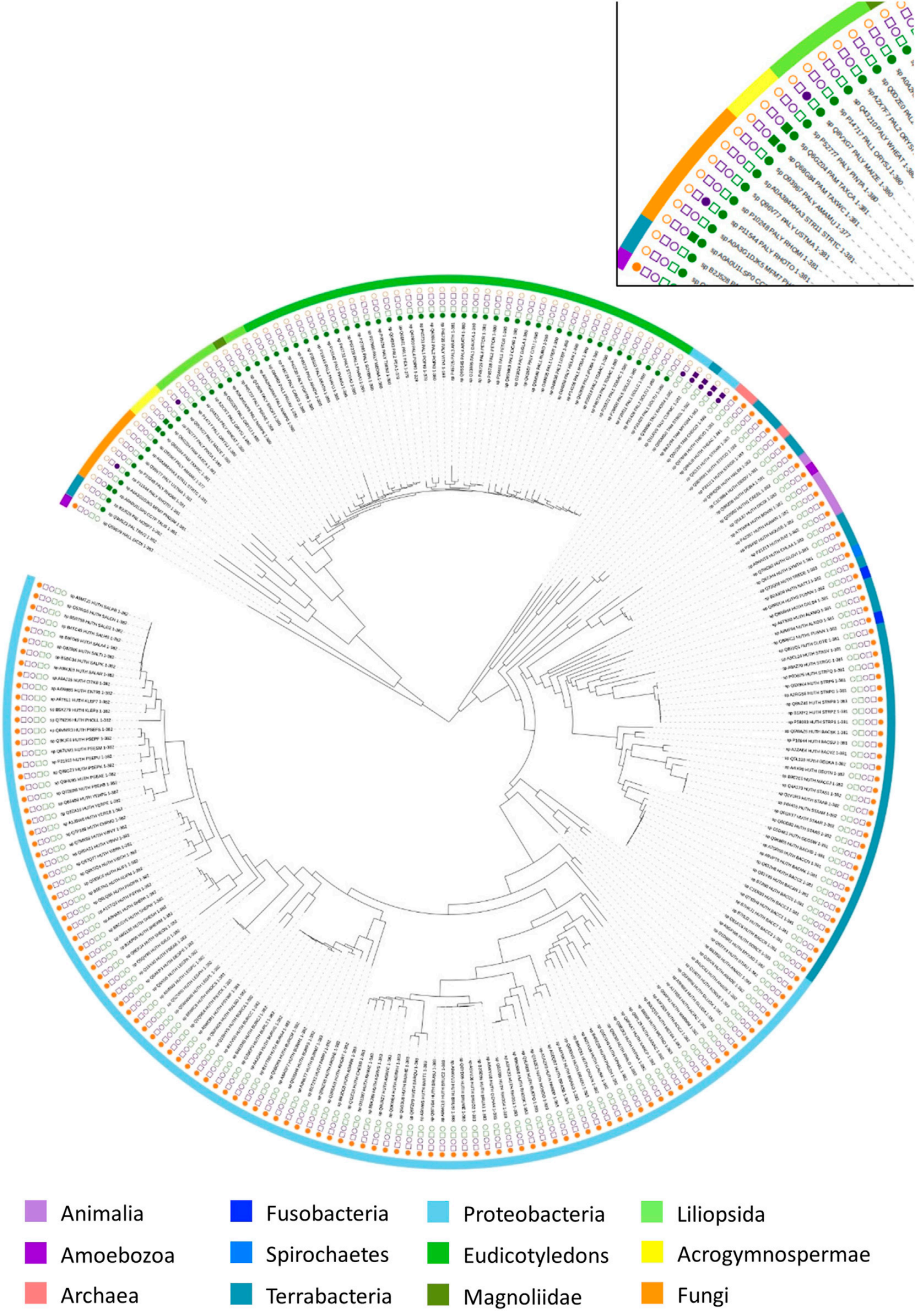
Phylogenetic tree of arylalanine amino acid ammonia-lyase and aminomutase homologs retrieved from the Swissprot database. Characterized ammonia-lyases are shown in the square while characterized aminomutases are shown in the circle. The color coding corresponds to the substrates of the enzymes: histidine (yellow), phenylalanine (green), tyrosine (purple). The source organisms in the tree are color-coded in the outer ring according to their origin.

To explore the evolutionary history of AAM, a phylogeny of 268 available biochemical-characterized protein sequences from the SwissProt was reconstructed (Bairoch et al., 2004). As displayed in Figure 5 and Supplementary Figure S1, AMM sequences from various phyla are grouped in two well-supported clusters (bootstrap support: 96%). One group contained all PAL sequences across different phyla and one eukaryotic HAL from *Dictyostelium discoideum*. The second cluster comprised all bacterial TALs/TAMs and the rest of the HALs. Overall, the phylogeny suggests that the arylalanine amino acids ammonia-lyase and aminomutase likely share common ancestry.

In the first group, the eukaryotic PALs represent a well-supported (100%) major phylogenetic branch with two groups in fungi and plants, which acquired the corresponding genes from a bacterial ancestor. The closest relatives of these sister groups are bacterial PAL from *Anabaena variabilis* and *Nostoc punctiforme* (91%). Both of these species are known to occur in diazotrophic symbiotic associations with fungi and plants that support the assumption of quite early genetic transfer of PALs from the bacterial ancestor to fungi and plants through an ancient symbiosis (Meeks et al., 2001). In contrast to PALs in bacteria and fungi, the phylogeny of the plant PALs diversified into three major sub-branches, including the *acrogymnospermae*, the *liliopsida*, and the *eudicotyledons*, which were described as the largest source of PALs. Among the plant PAL clusters, the acrogymnosperm clade was located between fungi and angiosperm and split into two mainly well-supported branches (100%) with monofunctional PAL from *Pinus taeda* in one branch as well as the PAL from genus *Taxus* (*Taxus canadensis* and *Taxus wallichiana* var. *chinensis*), which exhibits PAM activity as a side reaction, in the other branch. Furthermore, *D. discoideum*, as an obvious outsider among the eukaryotic HAL with the strong support of its position (100%), formed the deepest branching lineage in this large group and linked to the PALs clusters, indicating a probable single event of HGT from a prokaryotic ancestor in early age.

The second large group was further clearly separated into two branches with great statistical support (96%). One branch included all bacterial TALs/TAMs, which were found within the deep branching stable lineage, indicating that they were closer to the common ancestor than bacterial HALs. In the other branch, the genus *Streptomyces* (*Streptomyces avermitilis*, *Streptomyces coelicolor,* and *Streptomyces griseus*) developed a distinct bacterial HAL clade that divided off at the branching point from genus *Thermoplasma* (*Thermoplasma acidophilum* and *Thermoplasma volcanium*). Although this clade was located at a poorly supported position (44%), it still suggests that the HGT of the HAL gene could have occurred between the domains of Bacteria and Archaea. The phylogenetic tree shows the bacterial HAL mainly distributed into two large branches with all Proteobacteria in one branch and the *Terrabacteria*, *Fusobacteria*, and *Spirochaetes* in another branch. In the second branch, the eukaryotic HALs arose at an unsupported position, which was close to the bacterial clade of genus *Deinococcus*.

### Sequence Alignment

Usually, PALs and TALs have strict specificity for their natural substrates. Among them, the monofunctional TALs occur mainly in the bacterial kingdom though they are relatively rare in the archaea and eukaryotes. However, partial ammonia-lyases in fungi (*R. toruloides*) and monocots (*Zea mays*) are bifunctional with similar efficiency to phenylalanine and tyrosine. It is intriguing to assume the appearance of these PTALs by genetic transformation directly from bacterial TALs. Alternatively, as the bifunctional enzymes emerge in random species, this neofunctionalization event might be explained by mutations of residues in the enzymatic active center.

To explain the appearance of this bifunctional enzyme in plants and fungi, the sequence alignment was created with all enzymes with TAL activity and PALs in monocots from the phylogenetic tree (Figure 5). The carboxylate group of arylalanine amino acids is bound in a network of hydrogen bonds with the highly conserved residues of Asn247, Asn380, and Arg341 (in *Zm*PTAL) to stabilize the carbanion intermediate (Calabrese et al., 2004; Wu et al., 2012). The residues that surround the aromatic ring of amino acids (His123, Leu124, Tyr440, Lys443, Ile447, and Asp471 in *Zm*PTAL) are usually the key contributor to substrate selectivity (Supplementary Figure S2) (Baedeker and Schulz, 2002; Ritter and Schulz, 2004). Comparison of these residues from resulting multiple sequence alignments suggests that the *Rt*PTAL and *Zm*PTAL shared a higher degree of similarity in residue groupings with monofunctional PALs in monocots than bacterial TAL/TAMs. It supports the hypothesis that the bifunctional PTAL might originate in monocots by mutation and then were transferred through HGT to fungi.

Based on mutagenesis research and sequence alignment analysis, it is apparent that residues 137 and 138 strongly influence substrate selectivity in the AAM family. The His89Phe (His123 in *Zm*PTAL) mutation introduced the hydrophobic residue in TAL from *Rhodobacter sphaeroides*, resulting in complete substrate selectivity switch from tyrosine to phenylalanine (Watts et al., 2006).

## Discussion

### The Origin of AAMs and Mass Extinction Events

Interestingly, members of the AAM family usually are reported to play key roles in the production of radioprotective intermediates in both animals and plants. For instance, urocanic acid, the deaminated product from histidine by HALs, is a major epidermal chromophore that provides protection against UV-induced immunosuppression (Noonan et al., 1992). Similarly, in the plant kingdom, PALs are involved in the biosynthesis of flavonoids, which serve as DNA-protective metabolites against UV damage (Kootstra, 1994). Before the formation of the stratospheric ozone layer, ancient organisms suffered under higher UV exposure, which limited the possibility of land colonization (Hessen, 2008; Voosen, 2020). Thus, the ocean surface provided the primary protective shield for early marine organisms by adsorbing the most of the sun’s harmful ultraviolet radiation (Whitehead et al., 2000). The presence of the stratospheric ozone layer in the earlier Cambrian (around 600 million years ago) is thought to have influenced the divergence of multicellular animals, which led to the Cambrian explosion (Falkowski, 2011). The ozone layer served as a protective shield and promoted the colonization of land by higher plants and arthropods. The latter invaded and colonized the land during the Ordovician period, whereas the land plants appeared later (Rota-Stabelli et al., 2013). During land colonization, these organisms were exposed to a higher level of UV radiation than under water (Averof et al., 1995; Rubinstein et al., 2010), which presumably favored the evolution of the AAM gene in the common ancestors of early land dwellers. During this evolution of the related gene, the ancestors of vascular plants and terrestrial animals may have obtained or stimulated the ability to prevent damage from UV radiation.

The phylogenetic relations demonstrate that eukaryotic HAL probably evolved from prokaryotic ancestors earlier than plant PAL in multiple independent events, consistent with molecular time trees (Rota-Stabelli et al., 2013). Both arthropod and green algae are considered to be earlier colonizers of the terrestrial environment. Curiously, available genomic data indicate that the HUT pathway is missing in most lower metazoans and the arthropod, whereas the PALs are absent in red and green algae based on the available genomic data (Emiliani et al., 2009; Bender, 2012). The waxy cuticle of green algae and the exoskeleton or chitinous cuticles of arthropods might be considered to be various strategies to escape excessive UV exposure on the land. It can also be argued that AAMs did not evolve immediately after land colonization. The major extinction events were synchronous with volcanism. The volcanic gases resulted in ozone layer depletion, thereby elevating UV radiation on land (Lindström et al., 2019). Sculpture malformation in plant spores, spore tetrads, and pollen indicates the biological stress and evolutionary pressure on plants by increased UV radiation during the extinction interval (Benca et al., 2018; Marshall et al., 2020). Under high UV intensities, the frequency of this malformation increased in plant spores and pollen because of the DNA damage before the formation of the protective wall layer (Fields et al., 2020). Most immediately, ancient organisms, especially the ones that lived on land and in shallow water, were exposed to a high level of UV radiation during this period. As a strong mutagenic agent, UV irradiation might favor the appearance of AAM genes or at least the natural selection of organisms with AAM. Notably, the absence of the *pal* gene in terrestrial vertebrates and the disappearance of the HUT pathway in fungi and plants may also suggest a huge elimination of the old dominant species during the mass extinction event.

### Microbial Coevolution in the Early Terrestrial Ecosystem

Ancient bacteria faced the major challenge of limited natural resources and space and conflict with their neighbors within a community by the production of small antibiotic compounds. As the precursor of various antibiotics, β-tyrosine was generated through bacteria with TAM to compete against others. Based on the available genomic information, the representative bacterial species with TAL/TAM activity could be isolated from soil, for example, *Cupriavidus*, *Streptomyces,* and *Myxococcus*, which suggests that this gene arose in an early soil environment.

Except for the RsTAL from *Rhodobacter sphaeroides,* the other TALs exhibit tyrosine aminomutase activity with different preferred enantioselectivity, matching the configuration of their β-tyrosine-containing secondary metabolites: *(S)*-TAM from *Streptomyces globisporus* involves the synthesis of *(S)*-3-chloro-5-hydroxy-β-tyrosine in the antitumor antibiotic C-1027; In *Chondromyces crocatus*, TAM converts *(S)*-α-tyrosine to *(R)*-β-tyrosine for production of the cytotoxic chondramides (Rachid et al., 2007). Furthermore, the arrangement of enantioselectivity within the bacterial TAM clades reflects an independent evolutionary relationship, indicating that these traits of TAM may have evolved convergently (Krug and Müller, 2009). According to the most deeply branching line formed by RsTAL in the phylogenetic tree, it could be assumed that the ancestors of bacterial TAMs may have evolved from ancient bacterial TAL for the production of the chemical inhibitor and to impair nearby competitive organisms.

The *hutH* gene, encoding the cytosolic enzyme HAL, is considered an ancient and basal gene, participating in a core metabolism for the degradation pathway of l-histidine. It distributes broadly in bacteria and promotes them to utilize histidine as carbon and nitrogen sources (Fuchs and Kane, 1985). The loss of the HUT pathway in bacteria only leads to their lack of sufficient capacity for the utilization of histidine, but this is not lethal. Based on the phylogeny, the most coherent hypothesis for the HAL origin would be the HAL-like enzyme gene transfer from ancient TAL-bacteria to *Streptomyces* bacteria and/or thermophilic or halophilic archaea at quite early ages. Among the other archaea as well as among lower eukaryotes, the appearance of HAL is irregular and rare, whereas all vertebrates, especially mammals, require the *hutH* gene to metabolize histidine and suffer the disease caused by a deficiency of HAL (Ghadimi et al., 1962; Taylor et al., 1991). Of the protozoa HAL, it has so far only been assured in *D. discoideum*, which is identified as two homologs: the one placed in the eukaryotic HAL clade and the other one situated close to the cyanobacteria clade. It is noteworthy that the HUT pathway is absent from cyanobacteria. In cyanobacteria, the enzyme to catalyze phenylalanine conversion was the other member of AAM: PAL.

The bacterial PALs were rooted at the deepest position among the PAL clades, which is a clear indication of the original PAL emergence in bacteria. According to the evolutionary analysis of *Glomeromycotina*, it is proposed that the ancestral fungi have been found in symbiotic association with green algae or cyanobacteria before terrestrial colonization (Prasad et al., 2020). Nevertheless, neither HAL nor PAL orthologs in red and green algae were identified in the available genomic data. Besides this, the existing genomic information of bacteria and fungi with the *pal* gene, even the nearby amoebae with HAL activity, have a relatively high dependency on the soil environment, which might indicate that the gene transfer arose during coevolution between bacterial, fungi, and plants in the early terrestrial environment (Emiliani et al., 2009).

In most plant species, PAL is encoded by a multigene family, containing up to five members that express differently in numerous tissues or reply to various environmental stress conditions. For example, in *Arabidopsis thaliana*, *pal3* is expressed at a relatively lower level than the other three genes (*pal1*, *pal2*, and *pal4*) in stem tissue, whereas *pal3* seems to be mainly expressed in leaves, the most UV exposed plant tissue. Among these three genes, *pal1* and *pal2*, sharing the common promoter elements, mainly exist in roots and stems in biosynthesis processes related to abiotic imports, whereas the gene *pal*4 was expressed also in seeds (Raes et al., 2003). This gene duplication could be traced back to gymnosperms: *P. taeda* owns five *pal* genes, representing different tissues with various levels of expression (Bagal et al., 2012). It seems that plants received the *pal* genes from the bacterial ancestor and expanded mainly through gene duplication. Moreover, Shang et al. report that seven *pal* genes are tandemly arranged with four pseudogenes in two chromosomes from *Cucumis sativus* and point out that these *pal* genes were duplicated more recently as the splitting of cucumber from the other dicots (Shang et al., 2012).

**FIGURE 6:**
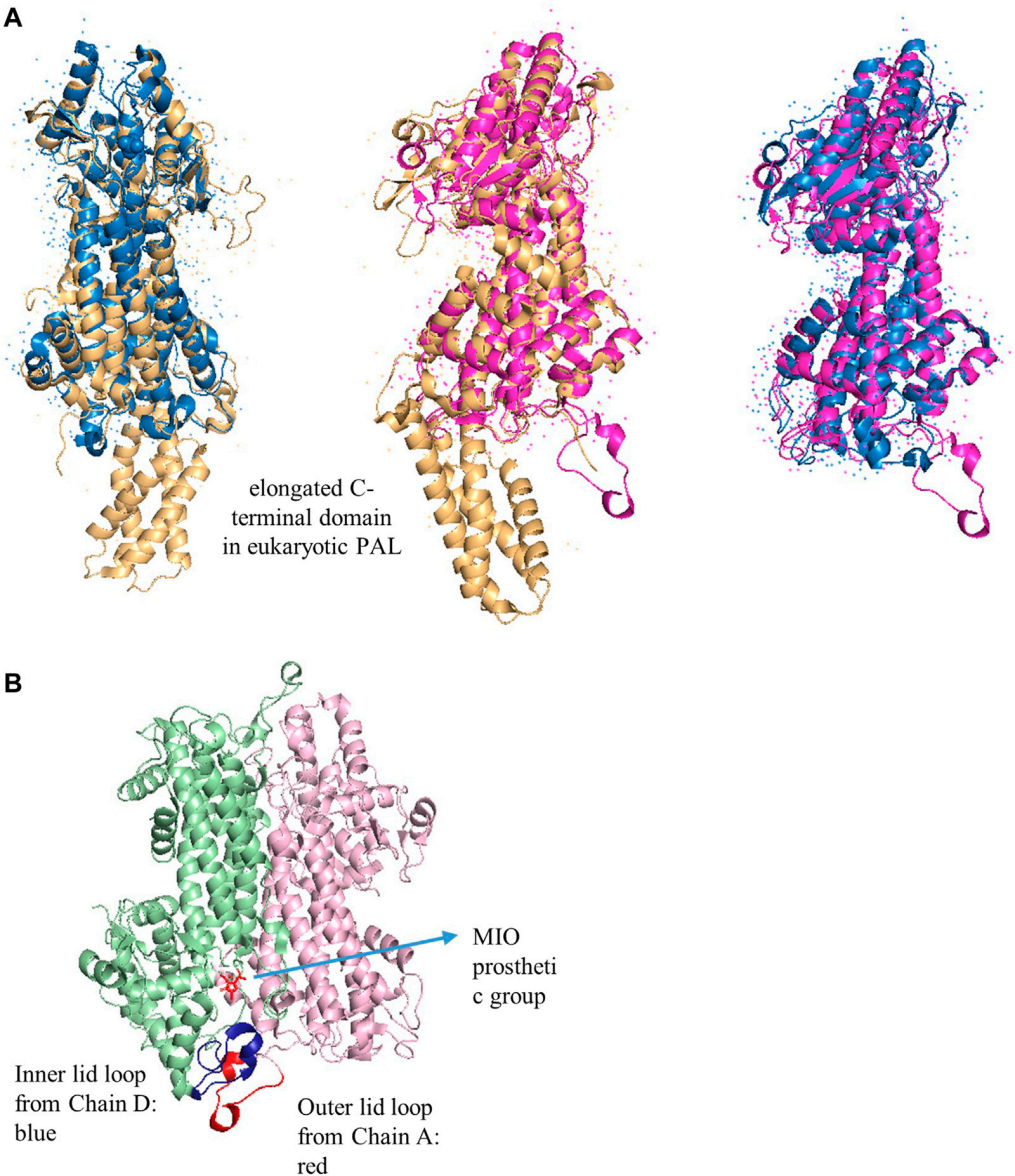
**(A)** Overlap X-ray crystal structures of PpHAL from *Pseudomonas putida* (PDB entry 1GKM, blue), RtPAL from *R. toruloides* (PDB entry 1T6J, yellow), and RsTAL from *Rhodobacter sphaeroides* (PDB entry 2O6Y, pink) **(B)** The inner (blue) and outer (red) loops in RsTAL from two chains. The MIO is colored in red.

Structurally, the quaternary sizes of the prokaryotic HALs (∼500 amino acids) and eukaryotic PALs (∼710 amino acids) from the AAM superfamily are diverse because of the presence of an additional C-terminal multihelix domain in the latter enzyme (Figure 6) (Allwood et al., 1999; Ritter and Schulz, 2004; Pilbák et al., 2006). These extended domains in plants are implicated to destabilize the enzyme for rapid regulation of the phenylpropanoid biosynthesis and adaptation of the varying environmental stresses. The phosphorylation site of PAL from *Phaseolus vulgaris* is determined as Thr545, whereas the most accessible cleavage sites by trypsin and chymotrypsin from *R. toruloides* PAL are identified as Arg123 and Tyr110, which are located in the extended regions to decrease the lifetime of PAL (Gámez et al., 2005).

### PTAL and PAM

PTAL, a specifical ammonia-lyase in monocots (especially the Poaceae family), is associated with the biosynthesis of stress-induced syringyl-rich lignins (Barros and Dixon, 2020). Among eight pal genes from the *Brachypodium distachyon*, only BdPTAL1 was expressed as the enzyme with additional TAL activity (Barros et al., 2016). The monocots with PTAL activity clustered with each other and were distinct from dicots. It can be assumed that this gene acquired an inverted substrate selectivity toward tyrosine after the early duplication event in monocots. The pattern of distribution of fungal and plant PTALs in the phylogenetic tree (Figure 4) and sequence alignment results (Supplementary Figure S2) might suggest that this gene originated in monocots and then transferred through HGT to fungi.

In other instances, yew species produce defensive metabolites, paclitaxel derived from β-phenylalanine, to protect themselves against widespread wood-degrading fungi (Malik et al., 2011). As long-lived species, yew trees are reasonably susceptible to fungal infection because they can form lateral buds on the old branches and the stem, which lead to a crash of barks (Thomas and Polwart, 2003). In these bark-cracking tissues, paclitaxel is mainly accumulated to hinder the pathogen attack (Talbot, 2015). The rate-limiting step of the side chain assembly process in the taxol biosynthesis is catalyzed by PAM that presumably evolved from the plant PAL ancestors in gymnosperms (Walker et al., 2004; Steele et al., 2005). Compared with the yew species, the other gymnosperms, such as *P. taeda,* employ the monofunctional PAL without any aminomutase activity. According to phylogenetic analysis, it is tough to conclude whether the PAL from *P. taeda* lost the catalysis ability as PAM during evolution in the early ages or the PAM gene in Taxus species comes from the ancient bacteria that obtained the PAM-like aminomutase. The taxol-producing endophytic fungi, including the *Penicillium* species, which tend to have PAL with aminomutase activity, could be isolated from the genus *Taxus* (Soliman and Raizada, 2013). Moreover, epiphytic and pathogenic fungi from the other hosts as well as saprophytic fungi are reported as taxol producers, especially the pathogenic fungi *Pestalotiopsis malicola*, which is isolated from soil, proposing another possible evolutionary scenario that points to a probable PAM origin from fungi (Bi et al., 2011). Based on this hypothesis, the old gymnosperms might obtain genes through HGT against fungal infection.

The dynamic simulation indicates that the inner loop is more conformationally flexible at higher temperatures or with more hydrophilic residues (A77T, I79S, C89T, and L97G in *Taxus chinesis* PAM), affording lyase features to aminomutase. Furthermore, the reaction temperature also influences the distance between phenolic O-atom of catalytic essential residue tyrosine and exocyclic methylene C-atom of the MIO prosthetic group in PcPAL from *Pelargonium crispum*, whereas HALs are highly tolerant to temperatures due to their stable inner loop (Pilbák et al., 2006; Heberling et al., 2015; Attanayake et al., 2018). Thus, the inner loop flexibility and environmental temperature are the determinants that distinguish the mutase vs. lyase activities. It is noteworthy that the growth rates of various fungal pathogens, like taxol producer *P. islandicum*, are significantly enhanced by the combination of warm temperatures and high humidity (Mannaa and Kim, 2018). It may indicate that the PAM emergence in plants is related to their protecting themselves against the pathogen fungi.

## Conclusion

The AAMs share a similar structure with highly electrophilic MIO and a common catalytic mechanism. Among them, eukaryotic PALs and HALs are involved in various metabolic and catabolic pathways to form several protection compounds under UV radiation. The corresponding genes most probably originated because of the fluctuating UV intensity. Our results indicate that the bacterial TALs have been developed and can produce antibacterial compounds under water-limited environments in the soils. Under UV exposure at an early age, some ancient bacteria were able to metabolize histidine by HAL or accepted phenylalanine as substrates by PAL, respectively. During further evolution, land plants and fungi obtained the PAL gene from bacteria through an early symbiosis while the terrestrial vertebrates inherited the HAL gene from their bacterial ancestor.

To conflict with the other organisms and withstand the pathogen infraction, the PAL gene was expressed in certain plants or fungi species as bifunctional PTAL or PAM. These corresponding genes were transferred through HGT between these two phyla. It may indicate that a symbiotic association involving bacteria, fungi, plants, and amoebae occurred in ancient terrestrial environments. Unlike PAM and TAM, there is no obvious genome evidence of the existence of aminomutase to produce β-histidine. It cannot be excluded that histidine aminomutase did not provide great advantages and, thus, disappeared during later evolution.

This review provides a phylogenetic framework for further evolutionary research of AAM, which has multiple meanings for studies of engineering in metabolic pathways and enzymatic biotechnology. Furthermore, more genomic data of new species and phylogenetic lines of this enzyme superfamily as well as substrate specificity data from well-investigated enzymes are also needed to find the missing puzzle pieces and integrate the evolutionary history.

## Methods

### Phylogenetic Analysis

To explore the phylogenetic history of arylalanine amino acid AAM, 268 available sequences were identified from SwissProt (Bairoch et al., 2004). These sequences were aligned using MAFFT version 7.3 (Katoh and Standley, 2013). After deleting the gaps within the resulting alignments by Gblocks (Talavera and Castresana, 2007), the 100% identified sequences were manually trimmed. The maximum likelihood trees were performed using the IQ-TREE version 1.4 (Minh et al., 2020) with the best-fit substitution model (LG + G) inferred by PhyML SMS servers (Lefort et al., 2017). Branch supports were assessed with 1000 bootstrap replicates and rooted using a minimal ancestor deviation (MAD) approach (Tria et al., 2017). The resulting phylogenetic tree was visualized with annotations by the online tool “interactive Tree of Life” (iTol) (Letunic and Bork, 2019).

### Sequence Analysis

The sequences of all TALs and certain PALs from monocots were identified from the SwissPort in UniProt knowledgebase (UniProtKB) and aligned with MAFFT version 7.3. Abbreviations and accession numbers from Swissport are RsTAL, *Rhodobacter sphaeroides* TAL (Q3IWB0); SgTAM, *Streptomyces globisporus* TAM (Q8GMG0); CmTAL, *Cupriavidus metallidurans* TAL (Q1LRV9); CcTAM, *Chondromyces crocatus* TAM (Q0VZ68); MfTAM, *Myxococcus fulvus* TAM (B8ZV93); RtPTAL, *R. toruloides* PTAL (P11544); ZmPTAL, *Zea mays* PTAL (Q8VXG7); BfPAL, *Bromheadia finlaysoniana* PAL (Q42609); NpPAL, *Narcissus pseudonarcissus* PAL (A0A2H5AIY6); OsPAL, Oryza *sativa subsp. Japonica* (P14717); TaPAL, *Triticum aestivum* PAL (Q43210).

## Data Availability

The data sets presented in this study can be found in online repositories. The names of the repository/repositories and accession number(s) can be found in the article/Supplementary Material.

## Supplementary Material

Supplementary Figure S1 Bootstrap tree of arylalanine amino acid ammonia-lyase and aminomutase homologs retrieved from Swissprot.

Supplementary Figure S2 Partial sequence alignment of eukaryotic PTALs, prokaryotic TAL/TAMs, and certain PAL in monocots. Shown is the location of the residues in the active center that are suggested to represent major roles for substrate selectivity. The specific residues in various domains are highlighted in different colors.

## Abbreviations

4CL, 4-coumaric acid-CoA ligase; AAM, Aromatic amino acid MIO-dependent enzyme; C3H, Cytosolic coumarate 3-hydroxylase; C4H, Cinnamic acid 4-hydroxylase; COMT, 3-O-methyltransferase; GFP, Green fluorescent protein; HAL, Histidine ammonia-lyase; HGT, Horizontal gene transfer; HUT, Histidine pathway; MIO, 3,5-dihydro-5-methylidene-4H-imidazole-4-one; PAL, Phenylalanine ammonia-lyase; PAM, Phenylalanine aminomutase; PTAL, Phenylalanine/tyrosine ammonia-lyase; PTM, Posttranslational modification; RFP, Red fluorescent protein; TAL, Tyrosine ammonia-lyase; TAM, Tyrosine aminomutase; UV, ultraviolet.
