# Supplementary figures and images for: Origin and Evolution of Enzymes with MIO Prosthetic Group: Microbial Coevolution After the Mass Extinction Event
Source: Front Genet. 2022 Mar 29;13:851738. doi: 10.3389/fgene.2022.851738 (PMC9002059; doi:10.3389/fgene.2022.851738)

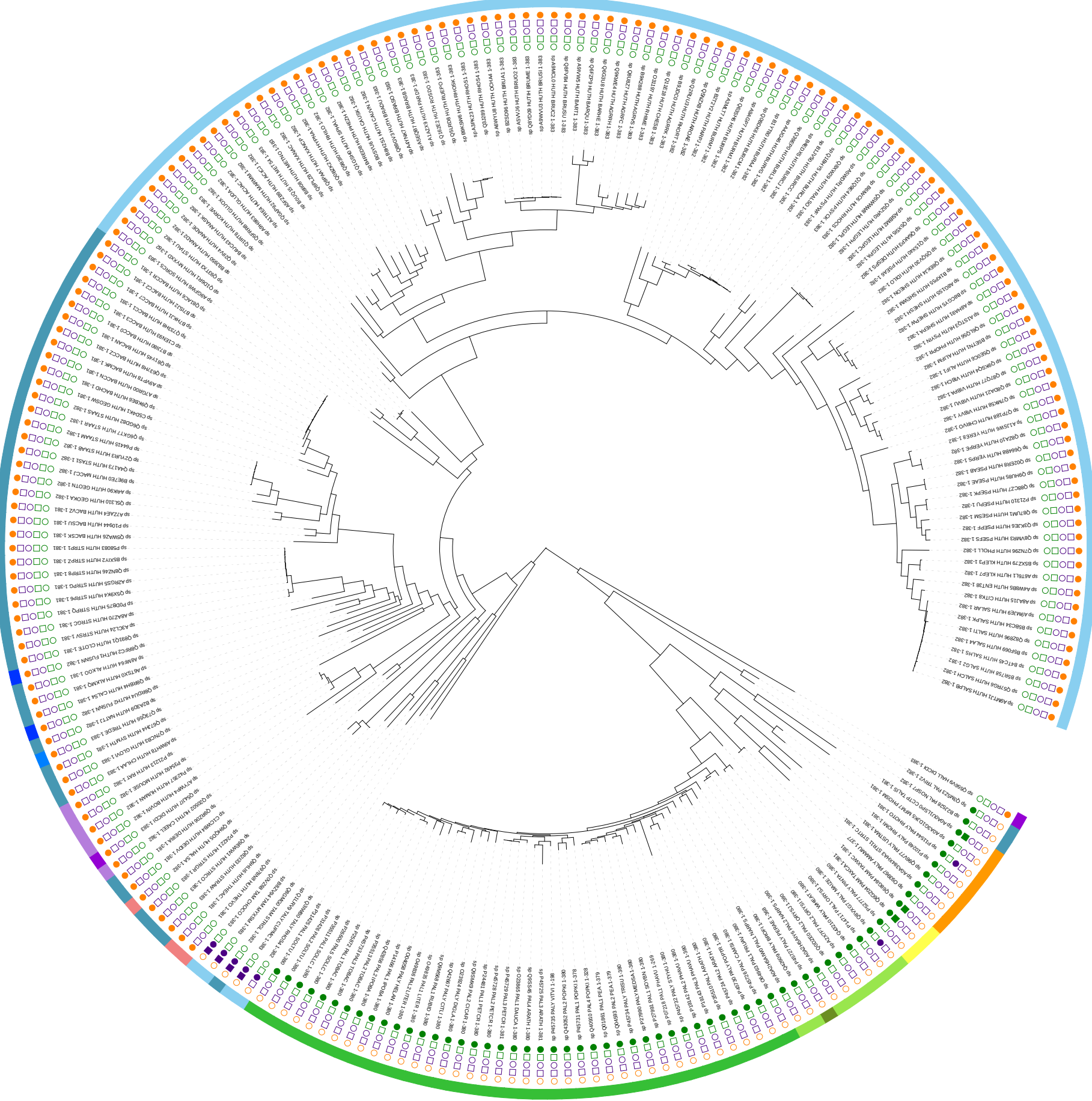

Supplement: Supplementary file 1 [file DataSheet2.PDF]

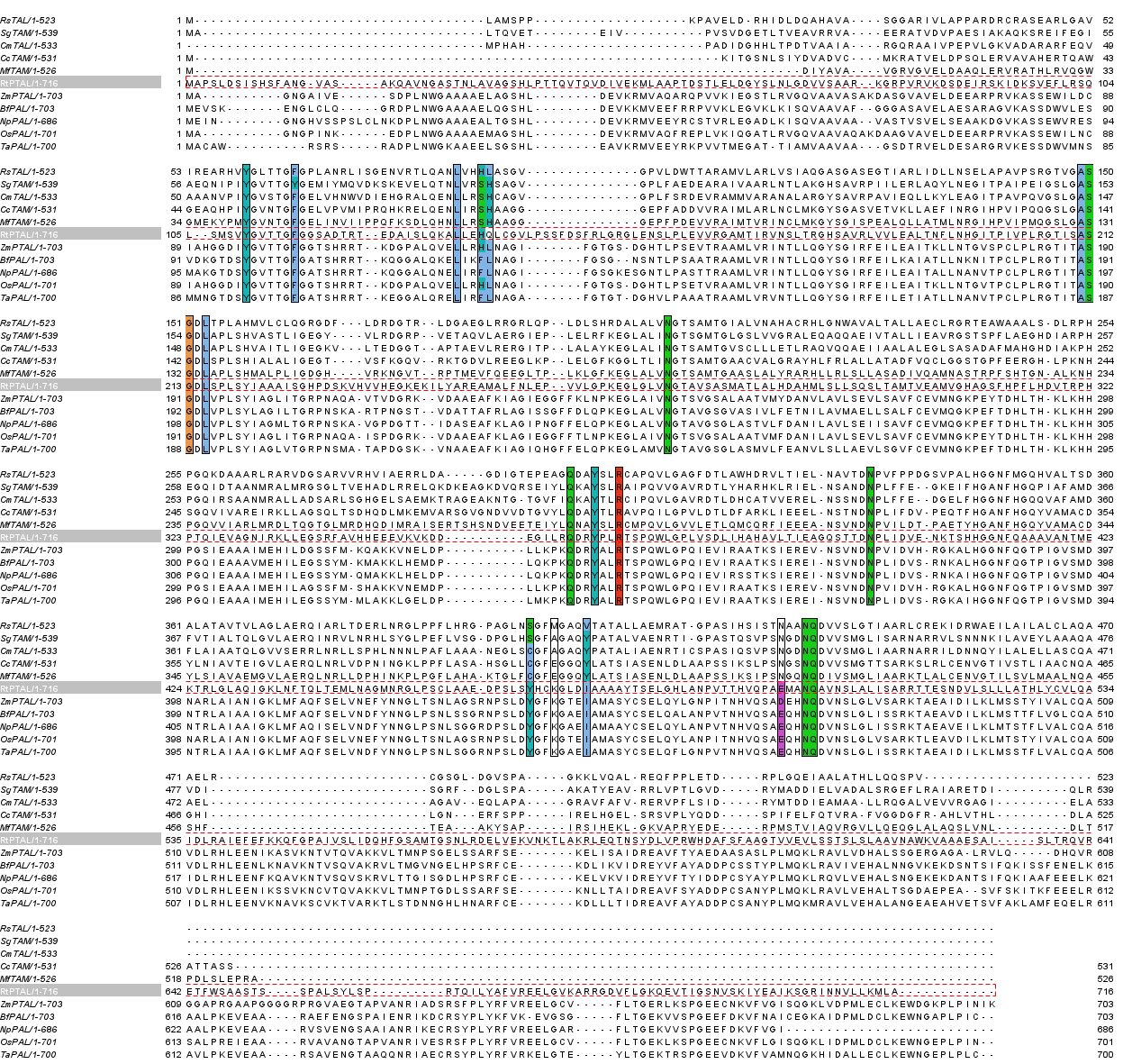

Supplement: Supplementary file 2 [file Image1.JPEG]

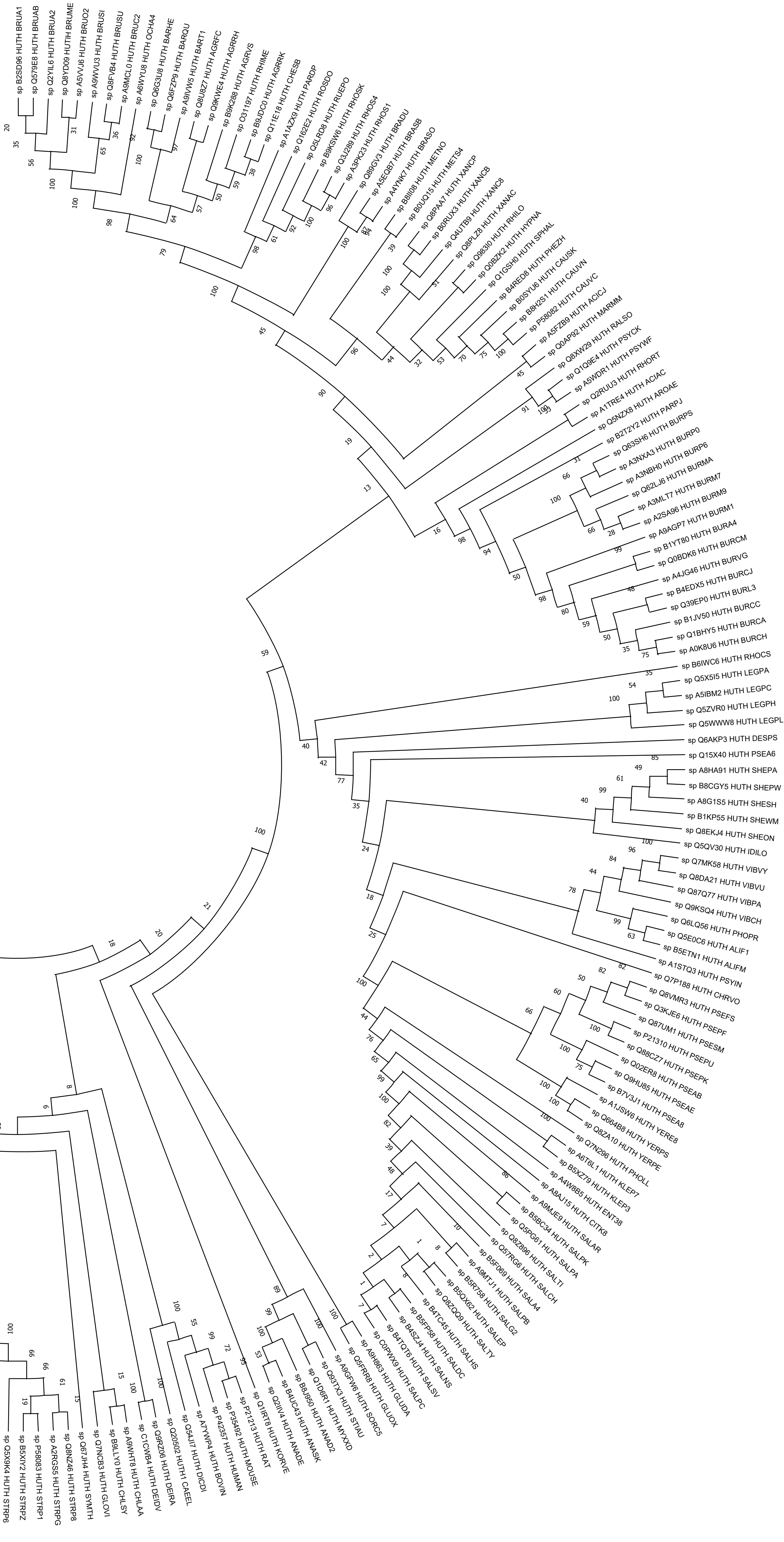

Supplement: Supplementary file 3 [file DataSheet1.PDF]
